# Supplementary material for: Salmonella Regulator STM0347 Mediates Flagellar Phase Variation via Hin Invertase
Source: Int J Mol Sci. 2022 Jul 30;23(15):8481. doi: 10.3390/ijms23158481 (PMC9368917; doi:10.3390/ijms23158481)

Figure S1. LC-MS/MS identification of FliC and FljB. (A and B) Protein sequences of FliC and FljB. **(C and D)** Intensities of specific peptides of FliC and FljB

A **FliC**

1 MAQVINTNSL SLLTQNNLNK SQSALGTAIE RLSSGLRINS AKDDAAGQAI
51 ANRFTANIKG LTQASRNAND GISIAQTTEG ALNEINNNLQ RVRELAVQSA
101 NSTNSQSDLD SIQAEITQRL NEIDRVSGQT QFNGVKVLAQ DNTLTIQVGA
151 NDGETIDIDL KQINSQTLGL DTLNVQQKYK VSDTAATVTG YADTTIALDN
201 STFKASATGL GGTDQKIDGD LKFDDTTGKY YAKVTVTGGT GKDGYYEVSV
251 DKTNGEVTLA GGATSPLTGG LPATATEDVK NVQVANADLT EAKAALTAAG
301 VTGTASVVKM SYTDNNGKTI DGGLAVKVGD DYYSATQNKD GSISINTTKY
351 TADDGTSKTA LNKLGGADGK TEVVSIGGKT YAASKAEGHN FKAQPDLAEA
401 AATTTENPLQ KIDAALAQVD TLRSDLGAVQ NRFNSAITNL GNTVNNLTSA
451 RSRIEDSDYA TEVSNMSRAQ ILQQAGTSVL AQANQVPQNV LSLLR

B **FljB**

1 MAQVINTNSL SLLTQNNLNK SQSALGTAIE RLSSGLRINS AKDDAAGQAI
51 ANRFTANIKG LTQASRNAND GISIAQTTEG ALNEINNNLQ RVRELAVQSA
101 NSTNSQSDLD SIQAEITQRL NEIDRVSGQT QFNGVKVLAQ DNTLTIQVGA
151 NDGETIDIDL KQINSQTLGL DSLNVQKAYD VKDTAVTTKA YANNGTTLDV
201 SGLDDAAIKA ATGGTNGTAS VTGGAVKFDA DNNKYFVTIG GFTGADAAKN
251 GDYEVNVATD GTVTLAAGAT KTTMPAGATT KTEVQELKDT PAVVSADAKN
301 ALIAGGVDAT DANGAELVKM SYTDKNGKTI EGGYALKAGD KYYAADYDEA
351 TGAIKAKTTS YTAADGTTKT AANQLGGVDG KTEVVTIDGK TYNASKAAGH
401 DFKAQPELAE AAAKTTENPL QKIDAALAQV DALRSDLGAV QNRFNSAITN
451 LGNTVNNLSE ARSRIEDSDY ATEVSNMSRA QILQQAGTSV LAQANQVPQN
501 VLSLLR

C

Intensity of FliC specific peptides

	WT	Δ*stm0347*
QINSQTLGLDTLNVQQK	1.71E+07	9.38E+05
VTVTGGTGK	6.72E+06	3.95E+05
NVQVANADLTEAK	3.67E+06	1.83E+05
AALTAAGVTGTASVVK	2.83E+06	1.47E+05
MSYTDNNGK	1.07E+07	2.59E+05
VGDDYYSATQNK	2.11E+06	8.84E+04
DGSISINTTK	4.25E+06	2.88E+05
LGGADGKTEVVSIGGK	4.57E+07	5.60E+06
TYAASKAEGHNFK	7.72E+06	1.08E+06
IDAALAQVDTLR	2.09E+07	1.33E+06
SDLGAVQNRFNSAITNLGNTVNNLTSAR	2.23E+07	6.90E+05

D

Intensity of FljB specific peptides

	WT	Δ*stm0347*
QINSQTLGLDSLNVQK	3.17E+05	2.15E+07
AYDVKDTAVTTK (+2)	1.28E+06	8.68E+07
AYDVKDTAVTTK (+3)	1.74E+06	8.68E+07
TTSYTAADGTTK	2.66E+05	1.24E+07
TEVVTIDGKTYNASK	1.21E+04	6.32E+05
AAGHDFKAQPELAEAAAK	4.16E+05	1.76E+07
IDAALAQVDALR	3.80E+05	1.31E+07
SDLGAVQNRFNSAITNLGNTVNNLSEAR	1.72E+05	7.81E+06
FNSAITNLGNTVNNLSEAR (+3)	2.16E+05	8.54E+06
FNSAITNLGNTVNNLSEAR (+2)	9.94E+05	3.26E+07

Figure S2. Swimming motility of WT and Δ*stm0347* strains under different viscosity conditions. Ficoll were added at the final concentrations of 0, 5 and 10% to the swimming plates. Data were presented as mean ± SD. n = 6. Asterisk indicates significant differences (*$p < 0.05$).

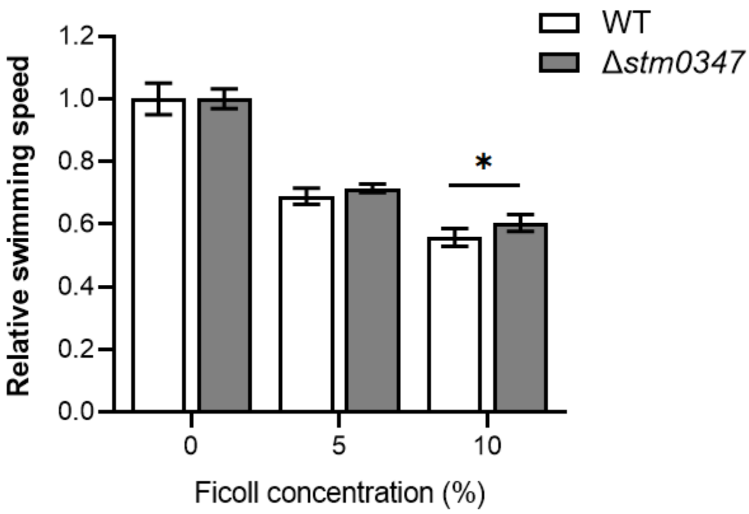

Supplement: Supplementary file 1 [file ijms-23-08481-s001.zip › Supplementary Figures.pdf]
